# The MasPar MP-1 As a Computer Arithmetic Laboratory

**DOI:** 10.6028/jres.101.018

**Published:** 1996

**Authors:** Michael A. Anuta, Daniel W. Lozier, Peter R. Turner

**Affiliations:** Cray Research Inc., Calverton, MD 20705; National Institute of Standards and Technology, Gaithersburg, MD 20899-0001; United States Naval Academy, Annapolis, MD 21402

**Keywords:** computer arithmetic, fixed-point and floating-point arithmetic, logarithmic and level-index arithmetic, residue number system arithmetic, serial and parallel simulation of computer arithmetic

## Abstract

This paper is a blueprint for the use of a massively parallel SIMD computer architecture for the simulation of various forms of computer arithmetic. The particular system used is a DEC/MasPar MP-1 with 4096 processors in a square array. This architecture has many advantages for such simulations due largely to the simplicity of the individual processors. Arithmetic operations can be spread across the processor array to simulate a hardware chip. Alternatively they may be performed on individual processors to allow simulation of a massively parallel implementation of the arithmetic. Compromises between these extremes permit speed-area tradeoffs to be examined. The paper includes a description of the architecture and its features. It then summarizes some of the arithmetic systems which have been, or are to be, implemented. The implementation of the level-index and symmetric level-index, LI and SLI, systems is described in some detail. An extensive bibliography is included.

## 1. Introduction

This paper describes and discusses the use of a massively parallel SIMD (single instruction, multiple data) computer system as a computer arithmetic laboratory. Specifically the Digital Equipment Corporation MasPar MP-1 computer^1^ with 4096 processors is used for software implementation of various types of computer arithmetic for integer, rational, real and complex arithmetic. The systems implemented (or, in some cases, to be implemented) include both conventional and novel number representations and arithmetic systems. Some of these provide general computational frameworks (such as binary integer and floating-point). Others have been developed primarily as special systems (such as the residue number system, RNS) or are still in experimental design stages (such as logarithmic, level-index and symmetric level-index arithmetic).

The first part of the paper contains a brief introduction to the MasPar architecture and why it is appropriate for this task. Section 3 reviews some of the number representations and their corresponding arithmetic data types which have been (or, in some cases, are being) created in this laboratory. In Sec. 4, we concentrate on one particular case. The implementation of the symmetric level-index, SLI, arithmetic serves as a particularly illustrative example of the general laboratory project because it uses some of the other arithmetic systems (such as fixed point fraction arithmetic of various wordlengths) for its internal processing. This section also contains details of a modified algorithm for SLI arithmetic which is better suited to a massively parallel implementation—and to an eventual VLSI hardware implementation of SLI arithmetic. A substantial bibliography is included.

## 2. The MasPar MP-1 System

The MasPar system is a SIMD array of 4096 processors configured as a square 64 × 64 array with toroidal wraparound in both directions. The individual processors are just 4-bit processors so that all arithmetic is implemented in software. Like any SIMD architecture, at any instant all processors are either performing the same instruction or are *inactive*. Clearly, for example, adding two 64 × 64 matrices is a particularly simple instruction for this machine. Matrix multiplication is less straightforward but is still well-suited to the array. Its speed advantage for such problems relative to conventional architectures comes from the massive parallelism overcoming the slower individual operations.

The principal advantages of using such a SIMD array for the implementation of a computer arithmetic laboratory arise out of its flexibility.

The 64 × 64 array of 4-bit processors can be used to simulate hardware implementations of the various arithmetic schemes and to make alterations easily in the algorithms being used. Alternatively the arithmetic can be implemented using serial algorithms so that the main computation is then spread across the processors. This will allow experimental computation to take advantage of the parallelism to reduce the time-penalty inherent in such a software system.

By implementing the standard floating-point and integer arithmetic in a similar manner, it should be possible to create a “level playing field” for comparing the performance of different arithmetic systems on particular problems. In particular, timing comparisons can be made with some justification since even the built-in arithmetic is “nibble-by-nibble.” A *nibble* is a half-byte, or 4 bits. Since a nibble corresponds to a hexadecimal digit, using radix 16 to implement the internal arithmetic of any system is natural.

The parallel array will allow realistic experimental computation without the enormous time-penalties which would be suffered on conventional serial machines—or even on pipelined vector processors.

By making a compromise between the “spread-the-arithmetic-across-the-array” paradigm and the “serial-algorithm-executed-in-parallel” alternative, speed-area tradeoff simulations can be run. The relative performances can be expected to be reasonably indicative of potential hardware, and so to alleviate the need for building experimental arithmetic units.

Many of these points will become clearer with reference to particular implementations. A later phase of the development of this computer arithmetic laboratory will be the simulation of various arithmetic hardware components. Then a prospective chip design could be mapped onto the array and tested.

The MP-1 supports programming in C and Fortran. The MasPar Programming Language (MPL) is an extended version of ANSI C allowing for *plural variables* which are variables for which there is an instance on each processor—or, more precisely in each processor’s individual memory. Communication between the various processors and their memories is achieved either through the *Xnet* (which is designed for neighboring communication in each of the North, South, East and West directions) or the *router* which handles more distant communications. The bandwidth of the Xnet is 16 times that of the router.

MPF (MasPar Fortran) is a version of high-performance Fortran, HPF, which again includes the appropriate array constructs and communication instructions. The two languages have been designed for the easy inclusion of subroutines written in one language within programs in the other. There is also a very powerful debugging and program-development environment which includes a profiler so that bottlenecks are easily identified.

## 3. Review of Proposed Computer Arithmetic Systems

Integer and floating-point arithmetic already exist in both MPL and MPF. Floating-point real and complex arithmetic is supported in the 32-bit and 64-bit IEEE (Institute of Electrical and Electronics Engineers) formats. Integers are supported in 8, 16, 32, and 64 bits in MPL, and in 32 bits in MPF. This section contains a brief summary of some of the other formats which are (or will be) available in the computer arithmetic laboratory. The list is merely illustrative and is not intended to be complete.

### 3.1 Integer and Fixed-Point Arithmetics

#### 3.1.1 Binary Integer Arithmetic

Binary integer arithmetic (two’s complement) already exists on the MP-1 and so need not be implemented specially for this laboratory. Indeed the shorter integer forms will be used as a basis for many of the other implementations. At a later stage of the development many of the hardware components of binary integer processors will be simulated to assist with the design of hardware algorithms. Details of these algorithms are readily available in standard texts such as Refs. [1–8]. Online algorithms, signed digit and redundant arithmetic (see Refs. [9–14] for example) are often used for internal computation. These would also be implemented during this later stage.

#### 3.1.2 RNS Arithmetic

Residue number systems (RNS) arithmetic has been extensively researched for well over twenty years and there is a very considerable literature on the representation, arithmetic algorithms and applications of such systems. A sample of these are listed in the Residue Number Systems section of the Bibliography, Refs. [15–29].

The principle of RNS arithmetic is that an integer within the representable range is represented by its residues modulo a set of basis primes. (Strictly, not all the basis elements must be prime but for most practical purposes this is needed.) Thus an integer *N* is represented in the RNS system using base moduli *p*_1_, *p*_2_, …, *p*_L_ by the vector (*a*_1_, *a*_2_, …, *a*_L_) where

$$a_{i}\equiv N\mathrm{mod}\;p_{i}\;(i=1,2,\ldots ,L).$$

Addition and subtraction of integers represented in this way can be performed by adding (or subtracting) the respective residues—and this may be done entirely in parallel since there is no carry from one modulus to another. The same is true for multiplication provided that the product does not overflow the *dynamic range*

$$M=\left(\prod_{i=1}^{L}p_{i}\right)-1.$$

(For many practical applications of RNS arithmetic, a symmetric range equivalent to [−*M*/2, *M*/2] would be used.)

The implementation of RNS arithmetic on the MP-1 would use one processor per modulus. Usually, the dimension *L* of the RNS-basis is much smaller than the 4096 processors available and so it becomes feasible to implement a degree of SIMD parallelism. For example even with a 64-dimensional RNS-basis, the MP-1 can simulate a SIMD processor with 64 processors each operating on this extended data type.

The implementation covers the common RNS integer arithmetic formats—both the nonnegative and symmetric forms. Conversion of either of these to binary integer forms can be achieved using the Chinese Remainder Theorem, CRT. The processor array can be used to implement the long accumulator which is needed for this conversion with a large dynamic range.

Other features which are included are base extension using a mixed radix conversion and the quadratic extensions of RNS integer arithmetic to admit complex integer arithmetic. Both the “real and imaginary part” form of the QRNS and the logarithm-based GEQRNS (Galois-enhanced quadratic residue number system) are implemented. (See Ref. [24] for example.)

Various RNS division algorithms have been (or will be) included for comparison purposes. These include the newer algorithms of Refs. [21] and [28]. One of the first applications of this arithmetic will be to the solution of linear systems and, in particular, the adaptive beamforming problem.

#### 3.1.3 Fixed-Point Fraction Arithmetic

One of the arithmetic forms which is often missing from the usual computational data types is fixed-point fraction arithmetic. Systems such as the lexicographic continued fractions of Kornerup and Matula [55–59] provide a general rational arithmetic. Otherwise, typically, binary fixed-point fractions are implemented as scaled versions of integers.

The fraction arithmetic implemented within this computer arithmetic laboratory allows direct computation with fixed-point fractions of varying wordlengths. Specifically, the wordlength is measured in “nibbles” (or hexadecimal digits). One nibble is reserved for sign and other information—such as a record of overflows for addition or the use of a reciprocation bit in division; see Sec. 4.1.

Fraction arithmetic is often required not only for itself but also for the internal computation of other arithmetic representations such as the level-index scheme which is discussed in greater detail in the next section. Some of the details of the implementation of fraction arithmetic are also presented there.

The use of the “nibble-base” means that multiplication of digits can be easily performed in an 8-bit integer format. Division is readily implemented using a radix-16 nonrestoring algorithm.

The basic fraction arithmetic is also to be extended for various library functions including some special function definitions which are needed for efficient algorithms for LI, SLI, or logarithmic arithmetic. These arithmetic algorithms also require the use of fixed-point number representations which have both an integer and a fractional part. These representations are accommodated by allowing “fractions” with *n.m* hexadecimal digits meaning *n* digits in the integer and *m* in the fraction.

### 3.2 Real Number Representations and Arithmetic

#### 3.2.1 Floating-Point Systems

The standard IEEE floating-point data types are already implemented in MPL and MPF. The laboratory will include software implementations of these with variations to allow for different wordlengths and different partitioning of those words between the exponent and mantissa.

For all the real number representations to be implemented, complex arithmetic will be implemented both in its conventional (real and imaginary part) form and in modulus-argument (or polar) form. Appropriate elementary and special function routines will also be available for each of these data types.

Much work has, of course, been done over the years on various aspects of the floating-point system. This has included the IEEE standards, hardware algorithm development, error analysis and correction, CORDIC (Coordinate Rotation Digital Computer) algorithms for elementary functions and multiple precision packages. (See Refs. [30–42], for example.)

Other variations on the basic floating-point arithmetic which are included are implementations of directed rounding so that interval arithmetic (Refs. [43–48]) may be simulated along with conventional arithmetic operations. In this context a “super-accumulator” for “exact” accumulation of floating-point inner products is to be implemented using the processor array to simulate the multiple precision unit.

The extended floating-point systems of Matsui-Iri [83] and Hamada [80,81,85] are based on the principle of only using the necessary number of bits in a floating-point word to represent the exponent. These are therefore developments of Morris’s tapered floating-point system [84]. The intention of both of these systems is to alleviate the overflow/underflow problem of floating-point arithmetic.

Matsui and Iri used part of the computer word to represent a pointer which indicates the number of bits allocated to the exponent with the rest then being available for mantissa representation. The relative representation error therefore grows with the magnitude of the number being represented, approximately linearly with the logarithm of its binary exponent. However, a “single precision” version of this representation requires 5 bits for this pointer and so can only yield higher precision over a very restricted range. The system is therefore suitable only for longer wordlengths.

This is also true of Hamada’s “Universal Representation of Real Numbers” or URR in which Matsui and Iri’s pointer is replaced by a dual purpose segment of the representation. In essence, this section of the word replaces both the pointer and the first bit of the exponent. Thus if the exponent has the form 2*^m^* + *n* the first bit is replaced by a unary string of *m* bits followed by a terminator. The rest of the exponent (the binary representation of *n*) occupy the next *m* bits and these are followed by the mantissa. Because of the need for the terminating bit in the representation of *m*, it follows that this representation is less compact than Matsui and Iri’s once *m* is greater than the pointer length of the latter representation.

The computer arithmetic laboratory will include both 32-bit and 64-bit versions of both these arithmetics as further variations on the binary floating-point system.

#### 3.2.2 Logarithm-Based Arithmetics

Logarithmic arithmetic has been extensively studied in recent years as an alternative to floating-point for real arithmetic. Work has included theoretical error analysis studies, algorithmic analysis and developments, and practical hardware processor designs. (See Refs. [49–54] for a sample of this work.)

The basis of logarithmic arithmetic is that a positive number is represented by its base 2 logarithm. This logarithm is represented in fixed-point form. The internal arithmetic of the logarithmic arithmetic in the MP-1 laboratory is therefore one of the places where the fixed-point binary fraction arithmetic referred to in Sec. 3.1.3. is used.

The recently developed algorithms based on polynomial interpolation techniques [53] will be incorporated into the implementation.

It is easy to extend the ideas of logarithmic arithmetic to an arbitrary base. Using *e* the base of natural logarithms may have some advantages for logarithmic complex arithmetic and for the evaluation of elementary functions within this system. This, too, will be added to the laboratory.

Natural logarithmic arithmetic is a bridge to the implementation of the level-index, LI, and symmetric level-index, SLI systems [60–79]. The implementation of these systems is discussed in greater detail in the next section.

## 4. SLI Implementation

Like many arithmetic systems the LI and SLI systems rely on a simpler arithmetic for their underlying internal arithmetic. In this case the underlying arithmetic is fixed-point fraction arithmetic. This section begins with a brief description of this and then of the LI and SLI implementations.

### 4.1 Fraction Arithmetic

In the fraction arithmetic of the MP-1 computer arithmetic laboratory, a number *f* with | *f* | < 1 is represented by a sign digit followed by a number of fraction digits. Each of these is a hexadecimal digit (or nibble) which simplifies spreading an arithmetic operation across the processor array.

The sign digit can obviously carry much more information than just the sign of the number. This additional space allows the storage of a reciprocation bit (or flag), and an overflow indicator bit. The reciprocation bit allows meaningful results to be returned for division of a larger number by a smaller one. If this result is itself to be used later as a divisor, unnecessary failure is thus averted.

Similarly, the “overflow bit” can be used to prevent overflow resulting from the addition of two fractions. In fact two such bits are available and these could be used to extend the representable range to (4, 4). Adding further integer nibbles can obviously extend this range.

Fractions of up to 15 nibbles can be stored in each processor using the MPL data type long long—a 64-bit integer which is one of its extensions of ANSI C. There are therefore packing and unpacking routines for conversion between types such as fraction10 (a fraction with sign plus 10 hexadecimal digits) and its various components. The bit manipulation operators of C make this operation reasonably straightforward. Further conversion routines are provided for changing between conventional real storage and the fraction types.

The available types will allow up to 15 hexadecimal digit fractions. Longer fractions can be stored by using an integer-type array in each processor—or, more likely, by using more than one processor. In either case multiple precision algorithms will be required to implement arithmetic operations.

Once the storage of such quantities is achieved, addition and subtraction are implemented by using their integer counterparts. The same is not true of multiplication.

Overflow (or wraparound) of integer multiplication is not appropriate since the most significant digits of the product are the ones which must be kept for fraction arithmetic. However the hexadecimal digit products can be constructed using unsigned 8-bit integer arithmetic and then combined with appropriate shifts to reformulate the result. Similarly hexadecimal digits provide a natural framework for a software radix-16 nonrestoring division algorithm.

The presence of the reciprocation bit necessitates a preprocessing of fractions for multiplication and/or division so that the correct sign and reciprocation sign are assigned to the result of the appropriate final arithmetic operation. For example division of a larger fraction, *x*, by a smaller one, *y*, is performed by setting the reciprocation bit of the result and computing the reciprocal quotient *y/x*.

Many of the design decisions here are reminiscent of those used in the Turbo Pascal implementation of SLI arithmetic described in Refs. [77–79].

### 4.2 LI Arithmetic

In the LI system a positive number *X* is represented by its *generalized logarithm x* where

X=ϕ(x). (1)

The generalized exponential function *ϕ* (the inverse of the generalized logarithm) is given by

$$\phi (x)=\{\begin{array}{ll} \;x & \text{if}\;0\leq x\leq 1,\\ e^{\phi (x-1)} & \text{if}\;x>1. \end{array}$$ (2)

The basic representation, arithmetic algorithms and analysis for this system were discussed in detail in Refs. [60–64,68,72].

To give a flavor of the MP-1 implementation of this system we describe just the algorithm for addition and subtraction, and its use of the fixed-point fraction arithmetic. This operation consists of finding *z* such that

ϕ(z)=ϕ(x)±ϕ(y) (3)

where *x* = *l* + *f > m* + *g* = *y* > 0 and *l* = [*x*], *m* = [*y*]. This is achieved by computing members of the sequences

$$a_{j}=\frac{1}{\phi (x-j)},b_{j}=\frac{\phi (y-j)}{\phi (x-j)},c_{j}=\frac{\phi (z-j)}{\phi (x-j)}.$$ (4)

The first two of these are evaluated by similar recurrence equations for *decreasing* values of *j*:

$$\begin{array}{cc} a_{j-1}=\exp(-1/a_{j}), & a_{l-1}=e^{-f},\\ b_{j-1}=\exp((-1+b_{j})/a_{j}), & b_{m-1}=a_{m-1}e^{-g}. \end{array}$$ (5)

The initial value for the *b*-sequence can be redefined to allow the simultaneous computation of these two sequences. Their values are bounded by 0 and 1 and the analysis of the algorithm [63] shows that they can be computed to fixed absolute precisions. It follows that fixed-point fractions are the desired internal arithmetic form.

The remainder of the algorithm consists of setting

$$c_{0}=1\pm b_{0},$$ (6)

then computing terms of the *c*-sequence by another short recurrence, and performing a final step to obtain *z*. The *c_j_*’s are included in [0, 1] for subtraction and [1, 2] for addition. Again, fixed-point fraction arithmetic is appropriate.

The analysis of the LI arithmetic algorithms [63] shows that, for a 32-bit LI wordlength, the data types fraction10 and fraction8 (that is fractions with 10 and 8 hexadecimal digits) are suitable for the computation of the *a*-sequence and the *b*- and *c*-sequences respectively. Furthermore, the sign nibble of the fraction representation above admits a 1-bit integer part so that the terms of the *c*-sequence for addition create no difficulty.

Efficient computation with these data types will certainly require implementation of special algorithms for the exponential and logarithm functions for the restricted range of arguments which are encountered in the LI algorithms. These special algorithms can be spread across the processor array. They would probably be based on the modified CORDIC algorithms originally presented in Ref. [75] or the table-lookup approach of Ref. [73]. (It is interesting to note that table-lookup has also been discussed in connection with logarithmic arithmetic in Refs. [53, 54].

Development of these algorithms is another task which will be eased by the computer arithmetic laboratory.

### 4.3 SLI Arithmetic

We begin with a brief description of a new SLI arithmetic algorithm and then consider its implementation in the MP-1 computer arithmetic laboratory. The notation here is the same as for LI arithmetic above except that now a real number *X* is represented by

$$X=\pm \phi {\left(x\right)}^{\pm 1}$$

with *ϕ* given by Eq. (2) and *x* ≥ 1.

#### 4.3.2 Modified SLI Algorithm

In the standard SLI arithmetic algorithms described in Refs. [63, 65] all the basic arithmetic operations involve the computation of a quantity *c*_0_ from which computation of the *c*-sequence proceeds.

For the “large” case, the add/subtract operation is just the LI operation in Eq. (3) above. Then *c*_0_ is given by

$$c_{0}=1\pm b_{0}=1\pm \frac{\phi (y)}{\phi (x)}.$$

The corresponding “mixed” operation is

$$\phi (z)=\phi (x)\pm \phi {\left(y\right)}^{-1}$$

with *c*_0_ given by

$$c_{0}=1\pm a_{0}\alpha_{0}=1\pm \frac{1}{\phi (x)\phi (y)}.$$

For “small” arithmetic the basic operation is

$$\phi {\left(z\right)}^{-1}=\phi {\left(x\right)}^{-1}\pm \phi {\left(y\right)}^{-1}$$

with *c*′_0_ = 1/*c*_0_ given by

$${c^{\prime }}_{0}=1\pm \beta_{0}=1\pm \frac{\phi (x)}{\phi (y)}.$$ (9)

There are similar recurrence relations to those in Eq. (5) which are used from appropriate starting values to generate the members of the *α* - and *β* -sequences given by

$$\begin{array}{c} \begin{array}{cc} \alpha_{j-1}=\exp(-1/\alpha_{j}j) & (j=m-1,\ldots ,1), \end{array}\\ \begin{array}{cc} \beta_{j-1}=\exp((-1+\beta_{j})/\alpha_{j}\beta_{j}) & \left(j=l-1,\ldots ,1\right) \end{array} \end{array}$$

where, again, *l*, *m* are the levels of *x*, *y* respectively. Note that in all cases, the first argument to the arithmetic operation is assumed to be the larger in absolute value so that *x* ≥ *y* for the large case and *x* ≤ *y* in the small case.

These arithmetic operations are analyzed in Ref. [65] in terms of the required precisions in the fixed-point computation of the sequences in order to deliver results with error comparable with inherent errors.

The alternative algorithms presented here are based on using only the *a*- and *α* -sequences. This has great potential advantages for both SIMD software and VLSI hardware implementation of SLI arithmetic since the definitions of these sequences are identical for the two arguments *x* and *y*.

These alternative algorithms reduce to redefining the initial values of the *c*-sequences by:

$$\begin{array}{cc} c_{0}=1\pm a_{0}/\alpha_{0} & \left(\text{large arithmetic}\right) \end{array}$$ (10)

$$\begin{array}{cc} c_{0}=1\pm a_{0}\alpha_{0} & \left(\text{mixed arithmetic}\right) \end{array}$$ (11)

and

$$\begin{array}{cc} {c^{\prime }}_{0}=1\pm \alpha_{0}/a_{0} & \left(\text{small arithmetic}\right) \end{array}$$ (12)

in place of Eqs. (7) to (9). The remainder of the algorithm remains unchanged. We observe here that the divisions in Eqs. (10) and (12) are always of a smaller quantity by a larger so that our fixed-point fraction arithmetic remains appropriate.

The precision requirements of the fixed-point internal computation will, of course, be slightly different for this modified algorithm. The detailed error analysis of this algorithm will be published elsewhere. The availability of variable wordlength fixed-point fractions will simplify computational testing of this algorithm.

Extensions of this algorithm to the extended arithmetic operations such as summation, scalar products and vector norm computations (see Refs. [69, 78] for example) yield further simplifications in the algorithm logic and therefore in the potential for VLSI hardware designs. A SIMD software implementation is a natural step in this direction.

#### 4.3.2 SLI Implementation

In this section we highlight some of the features of the MP-1 implementation of SLI arithmetic with reference to the task of summing a series of SLI terms which fits the processor array.

This example demonstrates some of the simplifications which follow from the adoption of the revised SLI algorithm described above. It is also a good vehicle for illustrating some of the features of the MPL language and its extensions of ANSI C. One of the primary benefits of this from the arithmetic viewpoint is that the SIMD instructions make it plain where there is multiple use of the same instruction which may be a good indicator of suitability for VLSI design. The many reduction algorithms that are built into the language also show clearly the places in a VLSI algorithm where adder, or other logic, trees would be used.

These advantages obviously carry over to any arithmetic system that is to be implemented on this or any similar SIMD architectures.

First the single precision, 32-bit, SLI data type slisingle can be identified with the 32-bit integer type long in such a way that the integer ordering is the correct SLI ordering. This is just the same data packing routine as was used in Refs. [77–79]. This order-preserving mapping is important for the identification of the largest element of the array of terms.

These terms would exist as a variable *X* of type plural slisingle which is to say it has one instance on each of the processors in the 64 × 64 array.

To describe the algorithm we shall denote the individual terms by

$$X_{i}=s_{i}\phi {\left(x_{i}\right)}^{r_{i}}(i=0,1,\ldots ,4095).$$

The largest element in this array of terms, and more importantly its position, can be obtained using the built-in MPL reduction functions reduceMax32 and rank32. We shall denote the position of the maximal element by *p*. For simplicity we shall assume |*X_p_* | ≥ 1 so that *r_p_* = 1.

The next step of the algorithm is to compute the *a*-sequence for each term. This operation is performed simultaneously on each processor to produce a plural fraction10 a[7] where again the word “plural” indicates the existence of this array on all processors. (The dimension 7 here reflects the maximum level needed in SLI arithmetic.) We shall denote the values of a[0] for the various operands by *A_i_*. Thus *A_i_* = 1/*ϕ* (*x_i_*).

The only branch in the algorithm is now used to compute the quantities

$$B_{i}=\{\begin{array}{ll} s_{p}s_{i}A_{p}/A_{i} & \text{if}\;r_{i}=+1,\\ s_{p}s_{i}A_{p}A_{i} & \text{if}\;r_{i}=-1. \end{array}$$ (13)

These terms are then summed over all processors to obtain c_0_ using the fraction equivalent of the built-in reduceAdd function. The number of terms demands that a maximum of 12 bits, or 3 hexadecimal digits, are needed for the integer part of *c*_0_.

The computation is completed by generating subsequent members of the *c*-sequence as for regular SLI addition.

The algorithm just described is much simpler than that presented in Ref. [78]. The use of the parallel instructions and reduction-based algorithms demonstrates clearly the inherent suitability of the algorithm for VLSI implementation.

The underlying fraction arithmetic requires just a few extensions beyond regular arithmetic operations. For example, a special purpose routine for computing exp(−1/*F*) for a fixed-point fraction *F* in (0, 1) to a fixed absolute precision is needed to compute the various *a*-sequences efficiently. This can be achieved using a modified CORDIC algorithm similar to those in Refs. [75, 78].

## 5. Conclusions

In this paper we have introduced the ideas behind the development of a software computer arithmetic laboratory on a massively parallel SIMD array processor. The particular machine used is a DEC/MasPar MP-1 with 4096 processors although the principles would apply equally well on any other similar SIMD machine.

A wide variety of number representations and arithmetic systems for computers can be incorporated into this laboratory. This paper has described some of those and then presented some salient details of just a few, including fixed-point fractions and the level-index and symmetric level-index systems. These systems and RNS arithmetic have been implemented while most of the others are yet to be added. Algorithmic improvements and modifications are being incorporated continually on the MasPar facility in the U.S. Naval Academy Mathematics Department.

The primary benefits to be gained are in the provision of a reasonable basis for comparison between various arithmetic forms and in allowing algorithmic experimentation as an aid to hardware design processes.

## Footnotes

1 Certain commercial equipment, instruments, or materials are identified in this paper to foster understanding. Such identification does not imply recommendation or endorsement by the National Institute of Standards and Technology, nor does it imply that the materials or equipment identified are necessarily the best available for the purpose.
